# EkoSonic™ Endovascular System-Directed Thrombolysis in a Patient With COVID-19 Infection Presenting With Bilateral Large Pulmonary Embolism Causing Right Ventricular Strain: A Case Report

**DOI:** 10.7759/cureus.21011

**Published:** 2022-01-07

**Authors:** Zahid Khan, Animesh Gupta, Umesh Kumar Pabani, Sunaina Lohano, Gideon Mlawa

**Affiliations:** 1 Cardiology, Royal Free Hospital, London, GBR; 2 Acute Internal Medicine, Barking, Havering and Redbridge University Hospitals NHS Trust, Romford, GBR; 3 Internal Medicine, Barking, Havering and Redbridge University Hospitals NHS Trust, Romford, GBR; 4 Geriatrics, Newham University Hospital, London, GBR; 5 Internal Medicine and Diabetes and Endocrinology, Barking, Havering and Redbridge University Hospitals NHS Trust, Romford, GBR

**Keywords:** low-molecular weight heparin, systemic thrombolysis, catheter-directed thrombolysis, morbidity and mortality, bleeding risk, pulmonary emboli, ekos catheter

## Abstract

We discuss a case of a 31-year-old male patient who presented to the accident and emergency department with shortness of breath and chest pain since the morning of the day of presentation. His polymerase chain reaction (PCR) test had returned positive for severe acute respiratory syndrome coronavirus 2 (SARS‑CoV‑2), which causes coronavirus disease 2019 (COVID-19), two weeks ago and his main symptoms had been shortness of breath, dry cough, generalized body pain, and fever. He was not vaccinated against the COVID-19 virus. He had not required hospitalization for COVID-19 and his symptoms had improved on day 10 from the date of diagnosis; however, he developed pleuritic chest pain with shortness of breath on the day of presentation. He was found to have tachypnoea, hypoxia, and tachycardia on assessment. His electrocardiogram showed a right bundle branch block with sinus tachycardia. He underwent a CT pulmonary angiography (CTPA) that showed bilateral large pulmonary emboli extending from the main pulmonary arteries bilaterally extending to the sub-segmental level. There was evidence of right heart strain on the scan. He also had a bedside echocardiogram performed after the CT scan, which showed an enlarged right ventricle but no left ventricular thrombus.

His blood results showed D-dimer levels of 14,000 ng/mL and troponin T of 255 ng/L. He received treatment with low molecular weight heparin (LMWH) and underwent emergency EkoSonic™ Endovascular System (EKOS) thrombolysis (Boston Scientific, Marlborough, MA). He remained on ultrasound-accelerated thrombolysis (USAT) for the next 12 hours and showed significant improvement and was taken off oxygen post-EKOS thrombolysis. He was discharged home on oral rivaroxaban after 48 hours of hospital stay; follow-up after two months showed normal-sized right ventricle with no evidence of pulmonary hypertension.

## Introduction

Pulmonary embolism (PE) is a common medical condition and is the third most common cause of cardiovascular death [1]. The prognosis of patients with PE depends on the degree of obstruction and hemodynamic effects of PE, and a certain group of patients develops the post-PE syndrome. Venous thromboembolism (VTE) is a major medical problem worldwide, particularly in hospitalized patients, with a total incidence of approximately 10 million cases per year. The annual incidence of VTE in hospitalized patients in the United States is approximately 600,000 and about 100,000 deaths are considered to be related to this, although the true incidence of VTE is not known [2].

EkoSonic™ Endovascular System (EKOS) (Boston Scientific, Marlborough, MA) or catheter-directed thrombolysis (CDT) is being widely used currently for patients with massive and submassive PE, and there are trials ongoing to assess its effectiveness versus thrombolysis [3]. The Pulmonary Embolism Response to Fragmentation, Embolectomy, and Catheter Thrombolysis (PERFECT) trial (2015), involving 101 patients with either massive or submassive PE who underwent EKOS, showed significant symptomatic and clinical improvement in right heart strain on echocardiography, decrease in pulmonary artery pressures, and minimal procedure-related complications [4].

Another prospective study, the SEATTLE II trial (2015), which involved 150 patients with massive and submassive PE who underwent EKOS, demonstrated significant improvement in right ventricular strain with a reduction in the right-ventricle-to-left ventricle (RV:LV) ratio and a decrease in post-procedure pulmonary hypertension [5]. The Ultrasound Accelerated Thrombolysis of Pulmonary Embolism (ULTIMA) trial (2014), conducted on 59 patients, showed significant improvement in right ventricular dysfunction in patients who received combination therapy with EKOS and heparin compared to those who received heparin alone, without any increase in complications risks [6].

## Case presentation

A 31-year-old male presented to the emergency department (ED) with shortness of breath and chest pain since morning. He had been diagnosed with coronavirus disease 2019 (COVID-19) two weeks ago when he had developed high-grade fever (39 °C), shortness of breath, and dry cough. He had no other past medical history and he worked as a chef in a restaurant. His body mass index (BMI) was 23 kg/m^2^. He was not on any regular medication, and his only significant family history was hypertension. He had started to feel better after a week and symptoms had improved significantly on day 10; however, he had still been feeling tired. He had not been very mobile due to his recent COVID-19 illness and had developed sudden-onset shortness of breath followed by left-sided pleuritic chest pain that was non-radiating. He had been apyrexial and presented to the ED.

On initial assessment, his blood pressure was 123/96 mmHg, heart rate was 112 beats per minute, respiratory rate (RR) was 24 breaths per minute, and oxygen saturation was 95% on 10 liters of oxygen. He had elevated D-dimer and troponin levels. His blood test results are shown in Table 1. He also underwent hepatitis and HIV testing for unclear reasons in the ED, which returned negative.

**Table 1:** Laboratory investigations results of the patient COVID-19: coronavirus disease 2019; PCR: polymerase chain reaction

Blood test	Normal value	Day 1	Day 2
Hemoglobin	133–173 g/L	126	128
White blood cell count	3.8–11 x 10^9^/L	9.85	8.19
Neutrophils	2–7.5 x 10^9^/L	8.96	11.37
Platelets	150–400 x 10^9^/L	294	303
Sodium	133–146 mmol/L	140	136
Potassium	3.5–5.3 mmol/L	4.6	4.4
Urea	2.5–7.8 mmol/L	5.8	9.7
Creatinine	59–104 umol/L	65	72
C-reactive protein	0–5 mg/L	4	5
Fibrinogen level	2–4 g/L	4.2	3.7
D-dimer	250–400 ng/ml	14,000	20,636
Troponin T	<14 ng/L	255	382
Hepatitis B virus surface antigen screen	Negative		
Hepatitis C virus antibody screen	Negative		
HIV 1 and 2 antibody level	Negative		
N-terminal pro-brain natriuretic peptide	56		
COVID-19 PCR	Negative		

**Figure 1:**
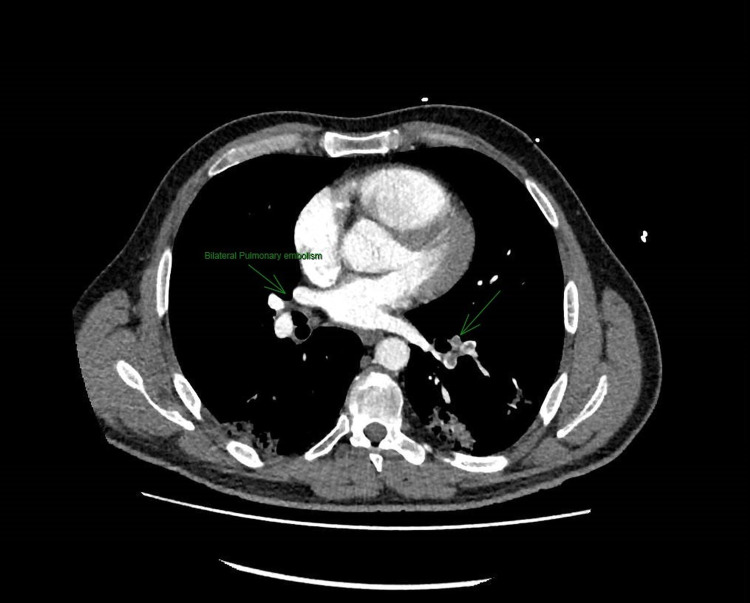
CTPA showing large bilateral pulmonary embolism CTPA: computed tomography pulmonary angiography

**Figure 2:**
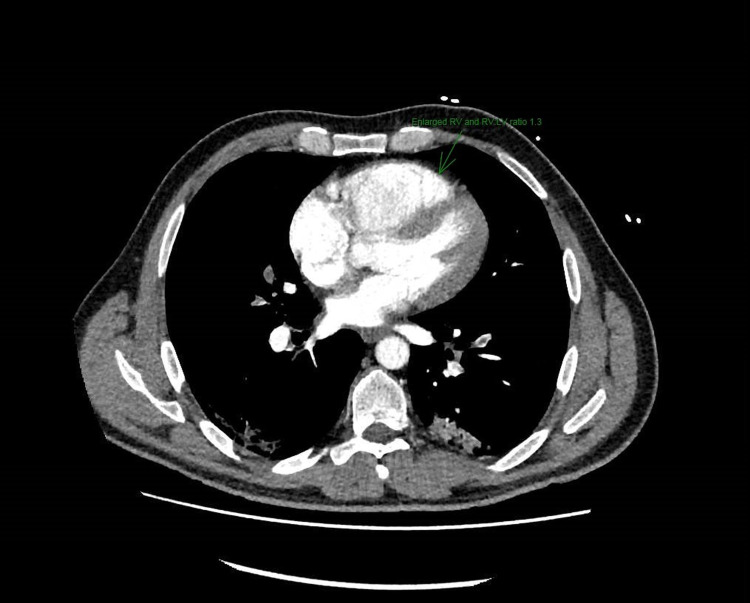
CTPA showing right ventricular strain and pulmonary embolism CTPA: computed tomography pulmonary angiography

The patient's COVID-19 polymerase chain reaction (PCR) test was negative and he was apyrexial. His ECG showed a right bundle branch block and he received a treatment dose of Tinzaparin [low molecular weight heparin (LMWH)]. His Chest X-ray was unremarkable apart from COVID-19 infiltrates. He then underwent CT pulmonary angiography (CTPA), which showed bilateral large pulmonary emboli extending from the main pulmonary arteries, seen with all lobes, extending to the sub-segmental level, and evidence of right heart strain with an RV:LV ratio of 1:3. The CTPA also confirmed bilateral patchy ground-glass attenuation in keeping with his COVID-19 infection (Figures 1, 2).

His PE Severity Index (PESI) score was 81 (Table 2).

**Table 2:** Pulmonary Embolism Severity Index (PESI) of the patient based on his comorbidities and investigations

PESI		
Age	31 years	31 points
Sex	Male	10 points
History of cancer	No	0 points
History of heart failure	No	0 points
History of chronic lung disease	No	0 points
Heart rate ≥110 beats per minute	No	0 points
Systolic BP <100 mmHg	No	0 points
Respiratory rate ≥30 breaths per minute	Yes	20 points
Temperature <36 °C/96.8 °F	No	0 points
Altered mental status (disorientation, lethargy, stupor, or coma)	No	0 points
O_2_ saturation <90%	Yes	20 points
Class II, low risk: 1.7–3.5% 30-day mortality in this group	Total score	81 points

**Figure 3:**
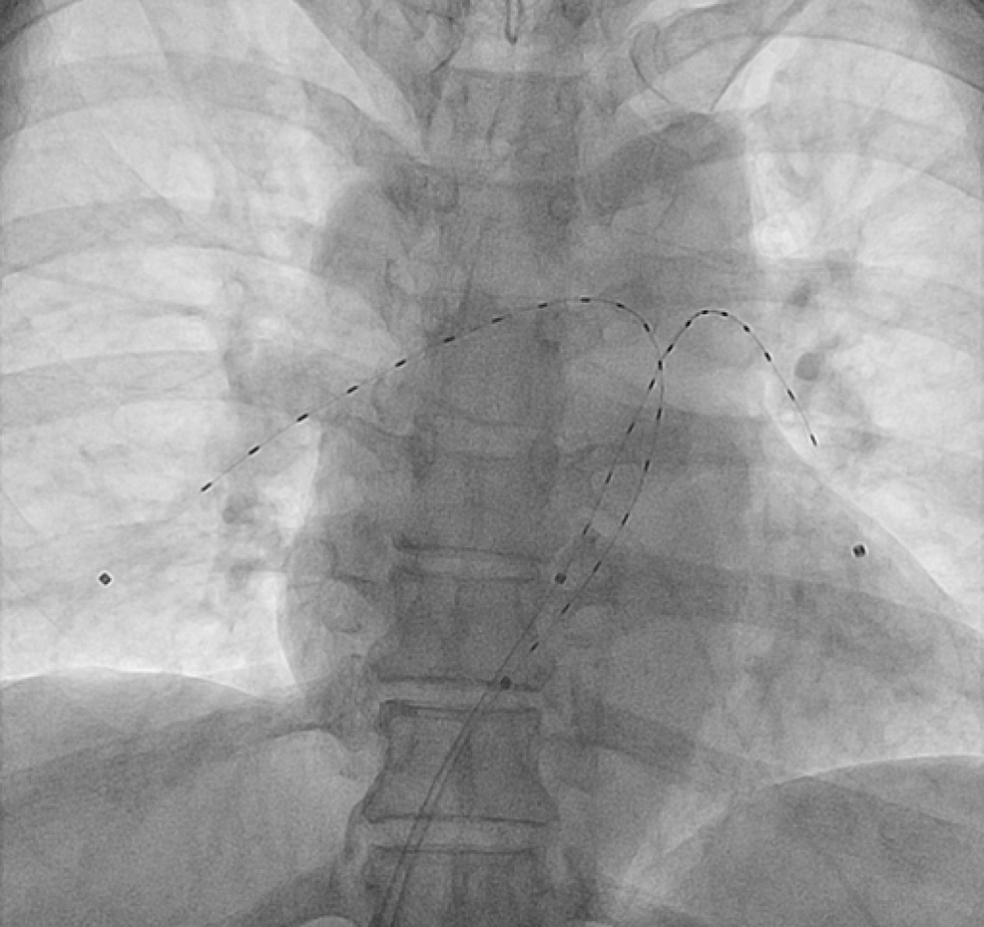
EKOS ultrasound image of the patient EKOS: EkoSonic™ Endovascular System

The patient had a bedside echocardiogram that showed an enlarged RV with no thrombus seen in the LV. He underwent an emergency EKOS-directed thrombolysis for large bilateral PEs. He was continued on alteplase infusion for the next 12 hours and his fibrinogen level was 3.7 g/L after three hours of the infusion. The EKOS sheath was removed after 12 hours and the patient received treatment-dose LMWH two hours after the removal of the sheath and was commenced on rivaroxaban 15 mg twice daily (BD) dose for 21 days initially, followed by 20 mg once daily (OD) for six months in total (Figure 3).

The patient showed significant improvement and his oxygen requirement dropped to 4 liters to maintain saturation of >96% eight hours after the procedure, and he had no oxygen requirement after 24 hours of the procedure. He was discharged home on oral anticoagulation 48 hours after the procedure, and a repeat echocardiogram two months later showed normal-sized RV and no evidence of pulmonary hypertension.

## Discussion

PE is a common medical condition, especially among hospitalized patients; however, its incidence among non-hospitalized patients has increased recently due to the ongoing COVID-19 pandemic [7]. The number of people who died from PE was 2,300 in 2012 in the UK, although the actual percentage of patients dying from PE went down by 30% [8].

EKOS is increasingly being used to treat patients with massive and submassive PE. A case report has described a 47-year-old female patient with a background of achondroplasia who was diagnosed with deep venous thrombosis (DVT) and PE; the patient underwent EKOS and achieved a satisfactory outcome [9]. The PERFECT trial showed that patients who had systemic thrombolysis for PE were more likely to develop complications and 20% of patients are likely to develop major bleeding, including a 2-5% risk of hemorrhagic stroke [4]. This trial included 53 men and 48 women, with an average age of 60 years. The trial showed clinical improvement in 24 of 28 patients with massive PEs and 71 of 73 patients with submassive PE, who received ultrasound- or catheter-guided thrombolysis. The mean pulmonary artery pressure showed improvement in most patients and follow-up echocardiography showed improvement in right-sided heart strain in 57 of 64 patients. CDT showed improved clinical outcomes in patients with acute PE while minimizing the risk of major bleeding, hemorrhagic stroke, or procedure-related complications [4].

The 2014 PEITHO randomized, double-blind trial involving 1,006 patients compared tenecteplase plus heparin with placebo plus heparin in patients with intermediate-risk pulmonary PE. Patients with acute right ventricular dysfunction and myocardial injury without overt hemodynamic compromise may be at intermediate risk for an adverse early outcome according to this study [10]. The study showed that CDT caused an increase in major bleeding and hemorrhagic stroke during their investigation of tenecteplase versus unfractionated heparin (UFH) in the intermediate-risk population. The study also reported that fibrinolytic therapy prevented hemodynamic decompensation in patients with intermediate PE but increased the risk of major hemorrhage and stroke. However, the 2015 PERFECT trial reported CDT to be associated with a lower risk of bleeding compared to systemic anticoagulation or LMWH [11]. Another study involving 63 patients, from 2009 to 2014, who underwent either CDT or ultrasound-accelerated thrombolysis (USAT) for massive or submassive PE showed that there was no statistical difference in clinical and hemodynamic outcomes or procedural complication rates between USAT and standard CDT [12]. CDT had the advantage of immediately decreasing the proximal thrombus burden in patients with massive or submassive PE, thereby reducing the afterload pressure on RV and improving cardiac output [13].

The 2014 ULTIMA trial, a randomized control trial, compared ultrasound-assisted CDT to intravenous heparin in the intermediate-risk population. The study reported that CDT was superior to anticoagulation in reversing RV dilatation compared to intravenous heparin [6]. The study did not report any increased bleeding risk or mortality risk in patients who underwent CDT at 90 days. Similarly, the 2015 SEATTLE II study, a prospective multicentre study, showed that CDT was more effective than conventional anticoagulation and it revealed decreased right ventricular dilation and lower long-term risk of pulmonary hypertension in these patients. It also showed reduced clot burden and no increased risk of intracranial bleeding in acute massive and submassive PE [5].

CDT is relatively safe, and the PERFECT registry showed only minor complications in 13 patients, which were related to minor bleeding events, and six of these were access site-related and none of these patients required blood transfusion. Similarly, the SEATTLE II study showed major bleeding in only 10% of all patients and only one patient had a significant groin hematoma requiring vasopressor support but none of the patients had intracranial bleeding.

The contraindications to CDT or USAT include patients with prior ischemic stroke, cerebral bleed, cerebral mass, and vascular deformation. Also, patients with recent gastrointestinal tract ulcers, recent major surgery, and any source of active bleeding are not considered candidates for CDT therapy [12]. It is important to be aware that dose adjustments may be necessary for patients with moderate risk of bleeding to prevent the above-mentioned complications.

## Conclusions

Our patient underwent emergency EKOS without any complications and we achieved a satisfactory outcome from the procedure. EKOS is becoming more popular and more centers will adopt it in the future for the treatment of massive and submassive PEs. It is relatively safe due to the fact that it offers local ultrasound-guided thrombolysis and the complication risk is relatively low compared to systemic thrombolysis. EKOS results in early patient discharge from the hospital and significantly reduces the risk of pulmonary hypertension and minimizes right ventricular dilatation, thereby reducing the morbidity and mortality risk.

EKOS thrombolysis, although effective, is not widely available and only certain centers are offering it at the moment in the UK. CDT offers improved clinical outcomes in patients with submassive or massive PE and it would be useful to conduct multi-center large-scale studies to prove its efficacy.
